# Pathogen and Testing Profiles in Post-Infectious Irritable Bowel Syndrome Across the COVID-19 Public Health Emergency: A Retrospective Case Series

**DOI:** 10.3390/jcm15197729

**Published:** 2026-10-08

**Authors:** Erhan Bozkurt, Osman Sağlam, Alper Sarı, Murat Ay, Oğuzhan Köse, Alper Emre Kurt, Ali Arda Demir, Mesut Çeliker

**Affiliations:** 1 Department of Internal Medicine, Afyonkarahisar Health Sciences University, Afyonkarahisar 03030, Türkiye; alpersari_@hotmail.com (A.S.); muratay09@gmail.com (M.A.); oguzhankose92@gmail.com (O.K.); dr.alper_emre@yahoo.com (A.E.K.); ali_arda_demir@hotmail.com (A.A.D.); mesut_celiker@hotmail.com (M.Ç.); 2 Department of Gastroenterology, Afyonkarahisar Health Sciences University, Afyonkarahisar 03030, Türkiye; ossag03@gmail.com

**Keywords:** post-infectious irritable bowel syndrome, COVID-19, gastroenteritis, diagnostic testing, antibiotics, disorders of gut–brain interaction

## Abstract

**Background/Objectives:** The COVID-19 public health emergency altered enteric infection surveillance, diagnostic testing, and antibiotic prescribing. We compared pathogen/testing composition among post-infectious irritable bowel syndrome (PI-IBS) cases stratified by whether index gastroenteritis occurred before, during, or after the emergency. **Methods:** This single-centre retrospective case series included 138 adults with clinician-diagnosed PI-IBS classified as Rome IV-compatible by chart adjudication. Index gastroenteritis occurred from January 2019 to December 2024; existing clinical records were reviewed retrospectively. The primary comparison was a five-category pathogen/testing profile. **Results:** The profile differed across pre-pandemic (*n* = 30), pandemic (*n* = 39), and post-emergency (*n* = 69) periods (χ^2^ = 35.56, *p* < 0.001; Cramér V = 0.36, 95% CI 0.30–0.48). Bacterial/protozoal cases represented 63.3%, 7.7%, and 33.3%, respectively; SARS-CoV-2-associated cases represented 0%, 35.9%, and 13.0%. Antibiotic exposure was 56.7%, 12.8%, and 33.3%. In an exploratory adjusted model, confirmed enteric pathogens were associated with antibiotic exposure (OR 16.28, 95% CI 3.03–87.35), whereas period was not. Sensitivity analyses excluding SARS-CoV-2 cases and period-crossing diagnoses were concordant. **Conclusions:** Pathogen/testing and antibiotic-exposure profiles differed across periods among diagnosed PI-IBS cases. These data describe case composition, not incidence or causal risk, and cannot separate infection ecology from diagnostic behaviour.

## 1. Introduction

Irritable bowel syndrome (IBS) affects approximately 4% of the global adult population when Rome IV criteria are applied and creates a substantial burden for patients and health systems [1]. Acute infectious gastroenteritis is one of its best-established environmental triggers. Post-infectious IBS (PI-IBS), defined as new-onset IBS after an acute enteric infection in a person without pre-existing IBS, develops in approximately 14.5% of patients after acute gastroenteritis and is associated with more than fourfold higher odds than in unexposed controls [2,3]. Risk differs by pathogen class and is generally higher after bacterial and protozoal infections than after viral infections [2,3,4]. Reported host and illness-related risk factors include female sex, psychological distress, acute-illness severity, and antibiotic exposure [3,4], although the association with antibiotic use was not confirmed in the most recent meta-analysis [2].

The COVID-19 pandemic disrupted the circulation and surveillance of enteric pathogens. Foodborne-disease networks documented marked reductions in reported bacterial infections during 2020–2021 [5], while giardiasis notifications declined substantially in several regions and remained suppressed into 2023 [6]. Altered susceptibility and exposure patterns after relaxation of public health measures have been discussed within the “immunity debt” framework [7]. In parallel, outpatient antibiotic prescribing and healthcare-seeking changed, and stool testing was variably affected [5,8]. SARS-CoV-2 also emerged as a plausible trigger for disorders of gut–brain interaction: prospective cohorts documented new-onset IBS after COVID-19, and meta-analytic evidence indicates a substantial PI-IBS association [2,9,10]. Population surveys have subsequently reported changes in the prevalence and burden of Rome IV disorders of gut–brain interaction [11,12].

Most available studies are population surveys, surveillance analyses, or cohorts defined by a single infection. Less is known about how the composition of patients ultimately diagnosed with PI-IBS changed within the same institution across the pre-pandemic, pandemic, and post-emergency periods. We therefore compared consecutive, chart-adjudicated PI-IBS cases according to the date of the index gastroenteritis episode. The primary objective was to compare a five-category pathogen/testing profile across periods. The key secondary objective was to compare antibiotic exposure during the index illness; IBS subtype and documented psychiatric diagnoses were exploratory. Because the study includes only PI-IBS cases and has no denominator of all acute gastroenteritis episodes, it cannot estimate PI-IBS incidence, relative risk, or causal effects.

## 2. Materials and Methods

### 2.1. Study Design, Setting, and Reporting

This single-centre retrospective case series with period comparisons was conducted in the Internal Medicine and Gastroenterology clinics of a tertiary university hospital in western Türkiye. The study was approved by the Afyonkarahisar Health Sciences University Clinical Research Ethics Committee (approval 2025/7; 7 February 2025), and the requirement for individual informed consent was waived because of the retrospective design. All index gastroenteritis episodes occurred between January 2019 and December 2024, before ethics approval. After approval, existing clinical records were reviewed retrospectively; the record review was completed on 20 September 2025, and subsequent IBS diagnoses documented in routine care up to that date were included. No patients were prospectively enrolled or followed. Reporting was organized in accordance with the STROBE recommendations [13].

### 2.2. Participants and Chart-Based Case Adjudication

**Figure 1:**
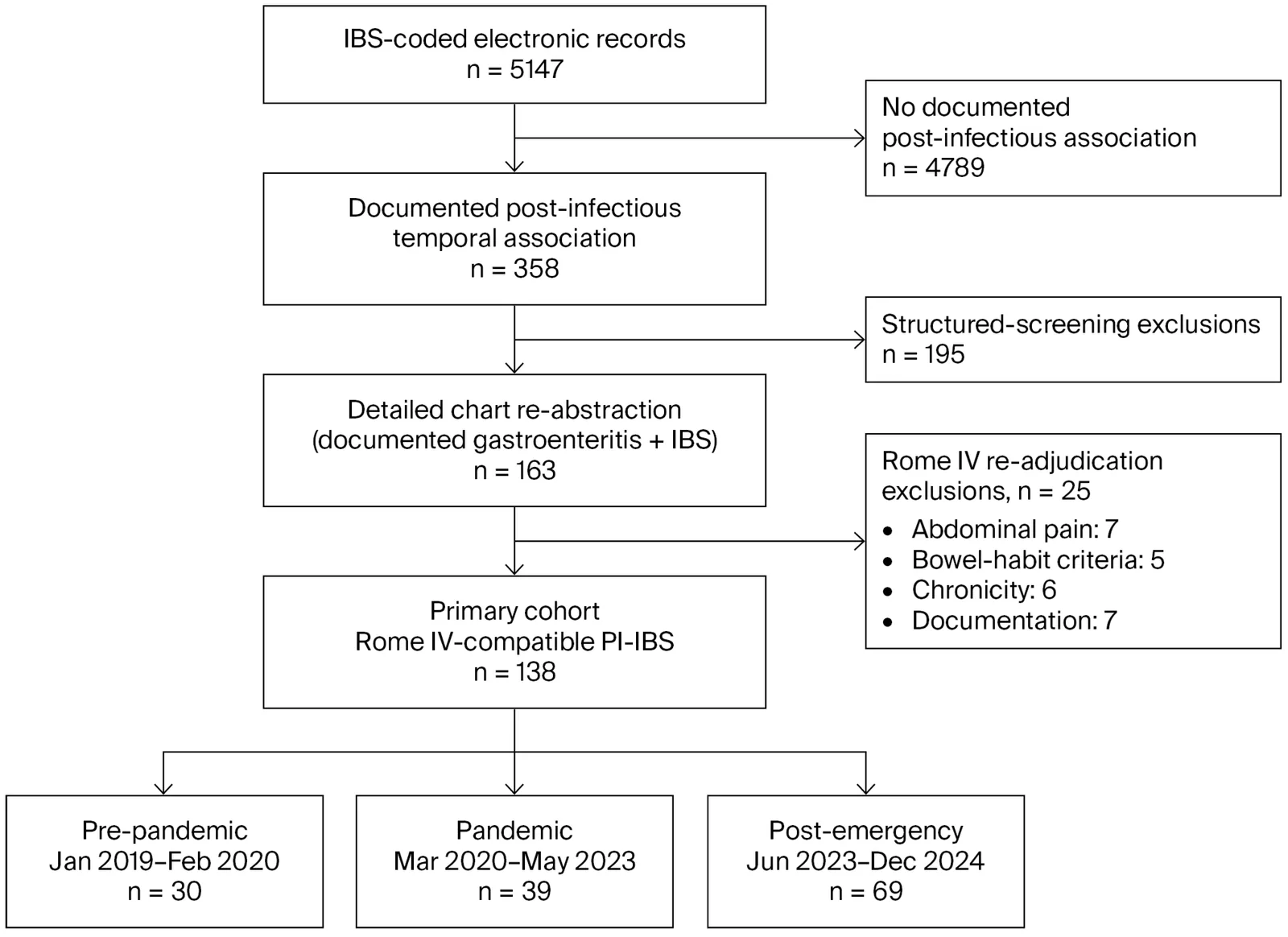
Patient flow. The 25 exclusions after Rome IV re-adjudication comprised recurrent abdominal pain criterion not met (*n* = 7), insufficient associated bowel-habit criteria (*n* = 5), chronicity criterion not met (*n* = 6), and insufficient documentation (*n* = 7).

An electronic health-record query returned 5147 records carrying an IBS diagnosis code. Records were screened for a documented preceding acute gastroenteritis episode with a temporal association to subsequent IBS symptoms (*n* = 358). Structured screening excluded 195 records: pre-existing IBS or another disorder of gut–brain interaction (*n* = 48), preliminary incompatibility with Rome IV documentation (*n* = 29), inflammatory bowel disease (*n* = 29), coeliac disease (*n* = 15), microscopic colitis (*n* = 9), gastrointestinal malignancy (*n* = 7), prior major gastrointestinal surgery (*n* = 9), age <18 years (*n* = 5), or insufficient records (*n* = 44). This left 163 records with a documented index gastroenteritis episode and clinician-diagnosed IBS for detailed re-abstraction (Figure 1).

New-onset status was established in two steps. At screening, records with IBS or another disorder of gut–brain interaction documented before the index gastroenteritis episode were excluded (*n* = 48). During detailed re-abstraction, the pre-episode record of each remaining patient was additionally reviewed for prior functional gastrointestinal diagnoses or chronic bowel-symptom documentation, and classification as PI-IBS required bowel symptoms newly documented after the index episode and persisting to the time of IBS diagnosis. Because this determination relied on retrospective documentation, undocumented pre-existing symptoms cannot be fully excluded, as noted in the Limitations.

Acute gastroenteritis was considered documented when a microbiological pathogen was identified or when at least two acute features (diarrhea, vomiting, or fever) were recorded, consistent with the Rome Foundation working-team framework [3]. Diarrhea and vomiting were recorded in all 163 records and fever in 71; the uniform recording of the first two features is addressed as a potential documentation artefact in the limitations. Classification as SARS-CoV-2-associated required laboratory-confirmed SARS-CoV-2 infection during the index illness, acute gastrointestinal symptoms fulfilling the gastroenteritis definition, and new bowel symptoms persisting beyond the acute infection. Classification was hierarchical: an identified enteric pathogen took precedence over SARS-CoV-2 status. However, no SARS-CoV-2-associated case had stool microbiology recorded during the index illness, so a concurrent enteric infection could not be microbiologically excluded in these 23 cases; this is acknowledged as a limitation.

Detailed chart re-abstraction included a binary adjudication of whether the retrievable documentation was compatible with Rome IV at the time of IBS diagnosis. Chart re-abstraction and Rome IV compatibility adjudication were performed jointly by two physicians—an internal medicine specialist (E.B.) and a gastroenterologist (O.S.)—using a structured abstraction template covering the documented pain and bowel-habit criteria, chronicity documentation, and exclusion diagnoses. Classifications were assigned by consensus during joint review. Records in which the available documentation was considered insufficient for a confident classification were not adjudicated as compatible but were excluded under the insufficient-documentation category. Because adjudication was performed by consensus rather than independently in duplicate, no inter-rater agreement statistic was calculated. Twenty-five records were classified as not compatible and excluded, leaving 138 cases in the primary analysis. Among the 25 records excluded after Rome IV re-adjudication, 7 lacked the required recurrent abdominal pain criterion, 5 had insufficient associated bowel-habit criteria, 6 did not meet the required chronicity criterion, and 7 had insufficient documentation for definitive Rome IV classification. Exact symptom-onset dates were not retained in the analytic dataset. Accordingly, the primary cohort is described as “Rome IV-compatible on chart adjudication,” not as a prospectively verified Rome IV cohort. The interval from index gastroenteritis to recorded IBS diagnosis was at least 211 days (median 278 days), but this interval is not equivalent to the Rome IV symptom-onset chronicity criterion.

### 2.3. Period Definitions and Study Variables

Cases were classified by the date of the index gastroenteritis episode as pre-pandemic (January 2019–February 2020), pandemic (March 2020–May 2023), or post-emergency (June 2023–December 2024). These periods spanned 14, 39, and 19 calendar months, respectively. Because IBS diagnosis could occur months after gastroenteritis, the diagnosis date crossed a period boundary in 27 of 138 cases; a sensitivity analysis was therefore restricted to the 111 cases whose index gastroenteritis and IBS diagnosis occurred within the same period.

The primary variable was a five-category pathogen/testing profile: bacterial (Salmonella, Campylobacter, or Shigella), protozoal (Giardia or Entamoeba), SARS-CoV-2, tested negative, or not tested. “Tested negative” denoted recorded stool microbiology without an identified pathogen, whereas “not tested” denoted no recorded stool microbiology. Stool-culture and parasite-examination indicators contributed directly to this operational classification and were therefore summarized descriptively but not analyzed as independent inferential outcomes.

Antibiotic exposure during the index gastroenteritis episode was recorded together with antibiotic class, monotherapy or combination treatment, timing relative to microbiology results, and duration. IBS subtype was classified as IBS with constipation (IBS-C), IBS with diarrhea (IBS-D), or mixed IBS (IBS-M) according to the recorded chart adjudication. Exact Bristol Stool Form Scale percentages were unavailable. Psychiatric comorbidity referred to a documented clinical diagnosis of anxiety and/or depressive disorder in the record, rather than a standardized psychometric instrument, and was categorized as pre-existing or newly documented after gastroenteritis. Demographic characteristics, fever, selected comorbidities, antidepressant exposure, and non-steroidal anti-inflammatory drug use were also abstracted.

### 2.4. Statistical Analysis

Continuous variables are presented as mean ± standard deviation or median (interquartile range), as appropriate; categorical variables are presented as *n* (%). Period-wise comparisons used Kruskal–Wallis tests for continuous variables and Pearson chi-squared or Fisher exact tests for categorical variables. The primary 3 × 5 pathogen/testing comparison used the Pearson chi-squared statistic. Because one expected cell count was <5 and SARS-CoV-2 was structurally absent from the pre-pandemic period, robustness was assessed with a fixed-margin Monte Carlo test based on 200,000 random contingency tables and with a sensitivity analysis excluding SARS-CoV-2 cases. Effect size was reported as Cramér V with a 95% percentile confidence interval from 10,000 stratified bootstrap samples.

Four secondary or exploratory period comparisons—antibiotic exposure, IBS subtype, any documented psychiatric diagnosis, and newly documented post-gastroenteritis psychiatric diagnosis—were included in a Benjamini–Hochberg false-discovery-rate family. Pairwise antibiotic comparisons used Fisher exact tests with Holm correction; absolute percentage-point differences were accompanied by Newcombe–Wilson 95% confidence intervals. An exploratory logistic regression modelled antibiotic exposure using period, confirmed enteric pathogen (bacterial or protozoal), fever, age, and sex. With 45 antibiotic-exposed cases and six slope parameters, this model was considered hypothesis-generating.

Sensitivity analyses evaluated: (1) the primary cohort after excluding SARS-CoV-2-associated cases; (2) cases whose gastroenteritis and IBS diagnosis occurred within the same period; (3) all 163 detailed-review records irrespective of the final Rome IV-compatible flag, using a four-category profile that combined tested-negative and untested cases; (4) the 68 microbiologically confirmed cases; and (5) a four-category confirmation analysis in the primary cohort combining tested-negative and untested cases. Analyses were performed using Python 3.13.5, SciPy 1.17.0, NumPy 2.3.5, and statsmodels 0.14.6. All tests were two-sided, with *p* < 0.05 considered statistically significant. ChatGPT-5.6 Pro (OpenAI) was used solely for language editing and improving the clarity and organization of the manuscript. The authors critically reviewed and verified all outputs and take full responsibility for the final content. Generative AI was not used for data generation, statistical analysis, interpretation of results, or drawing conclusions.

## 3. Results

### 3.1. Cohort Characteristics

The primary analysis included 138 chart-adjudicated Rome IV-compatible PI-IBS cases: 30 pre-pandemic, 39 pandemic, and 69 post-emergency (Figure 1). Ninety-three participants (67.4%) were women, the mean age was 40.8 ± 14.2 years, and the median gastroenteritis-to-diagnosis interval was 278 days (IQR 257–299). Age, sex, interval, diabetes, hypertension, antidepressant exposure, and NSAID use did not differ across periods (Table 1). Fever documentation varied across periods (66.7%, 23.1%, and 49.3%; *p* = 0.001), consistent with the differing aetiologic composition.

### 3.2. Pathogen/Testing Profile

**Figure 2:**
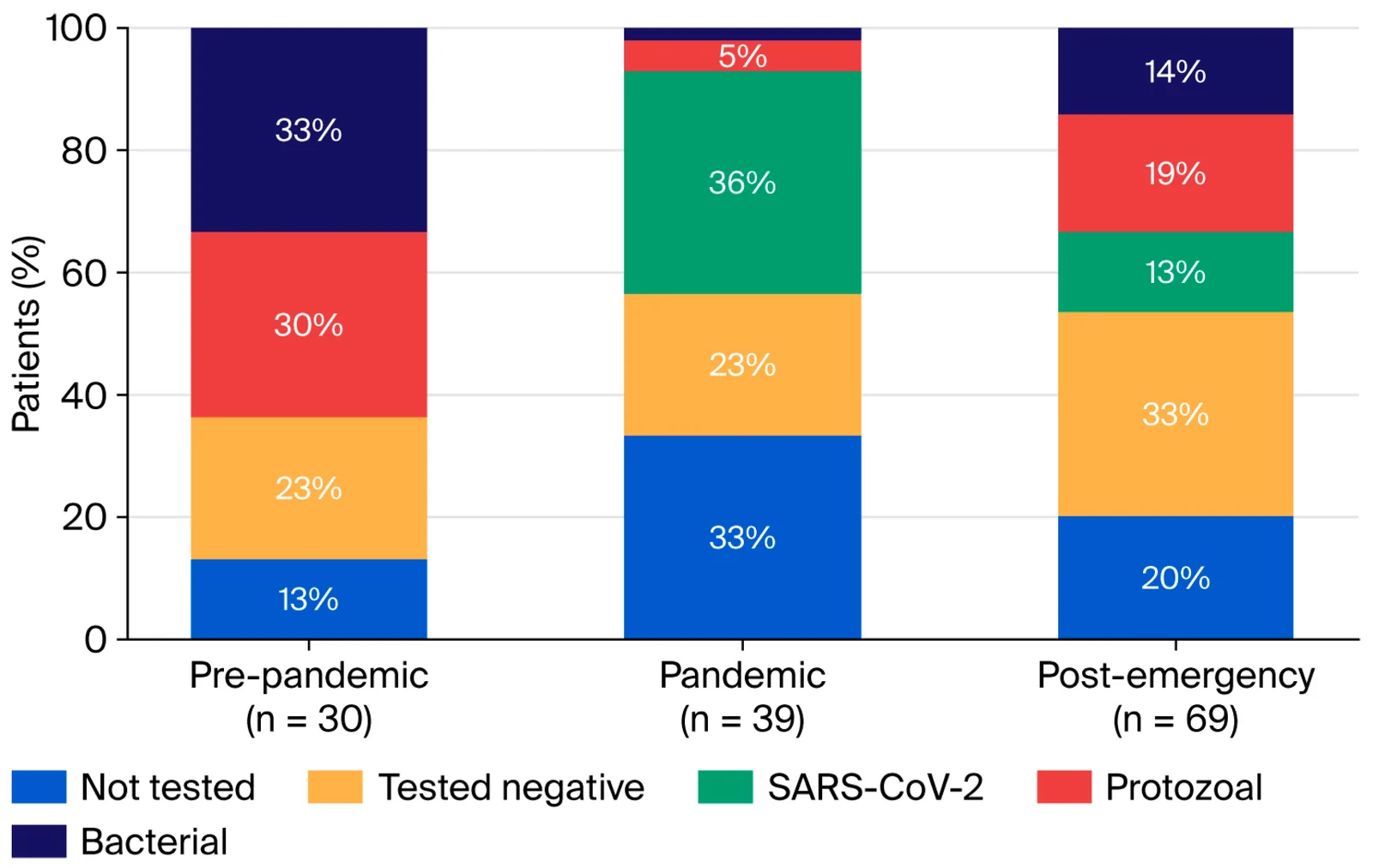
Five-category pathogen/testing profile among primary-cohort cases by index gastroenteritis period. Bars show the percentage distribution within each period.

The five-category pathogen/testing profile differed across periods (χ^2^ = 35.56, df = 8, asymptotic *p* = 0.000021; fixed-margin Monte Carlo *p* = 0.000030; Cramér V = 0.36, bootstrap 95% CI 0.30–0.48; Table 2 and Figure 2). Bacterial plus protozoal cases represented 63.3% of pre-pandemic cases, 7.7% of pandemic cases, and 33.3% of post-emergency cases. SARS-CoV-2-associated cases represented 35.9% during the pandemic and 13.0% during the post-emergency period. The proportion of cases that were not tested was 13.3%, 33.3%, and 20.3%, respectively, while tested-negative cases accounted for 23.3%, 23.1%, and 33.3%. The four-category confirmation analysis combining tested-negative and untested cases was concordant (χ^2^ = 32.52, df = 6, *p* < 0.001; V = 0.34; Supplementary Table S3). The distribution of individual organisms by period is given in Supplementary Table S7; Giardia was the most frequently identified single organism (19 cases).

### 3.3. Antibiotic Exposure and Case Mix

**Figure 3:**
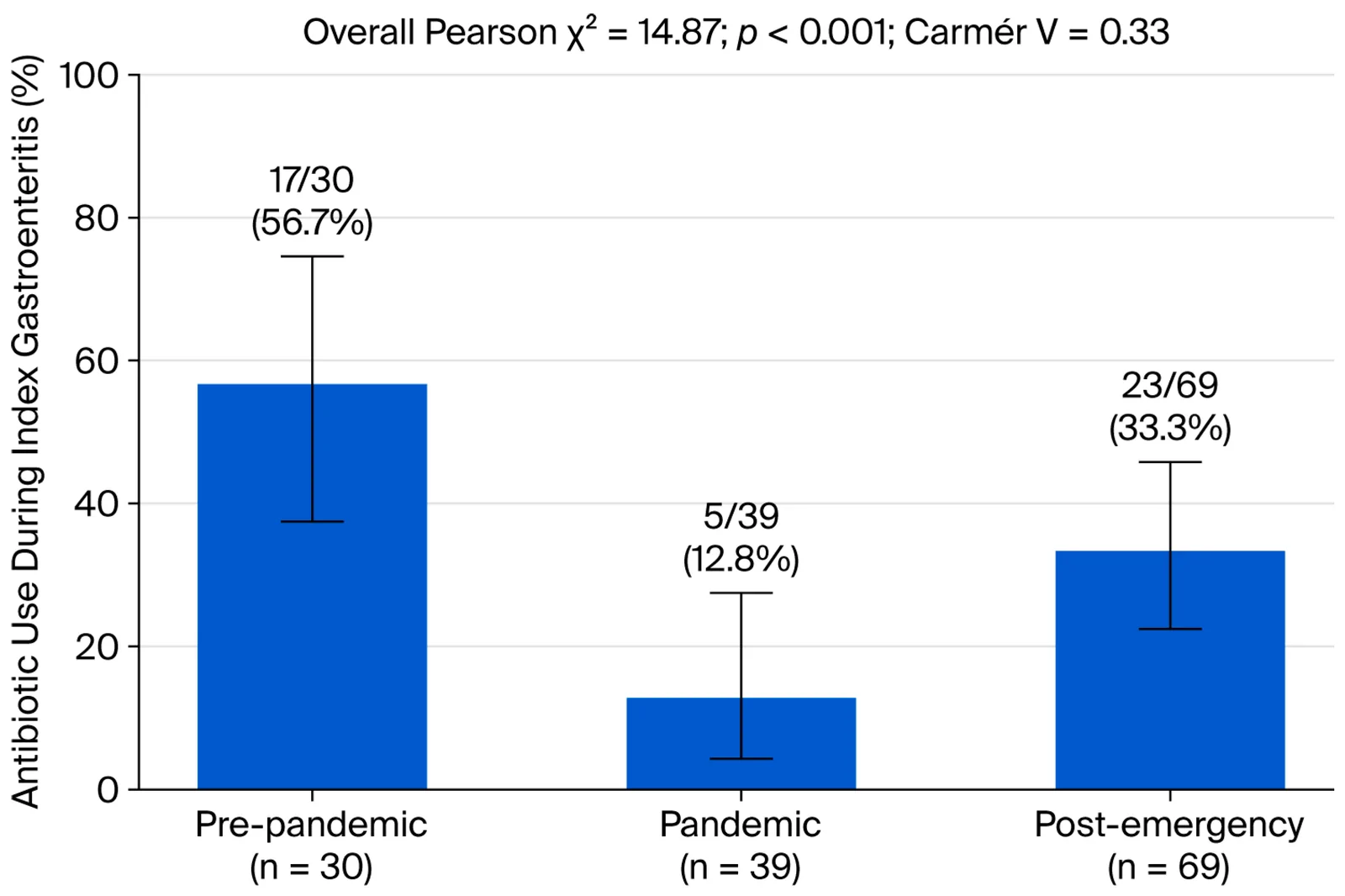
Antibiotic exposure during the index gastroenteritis episode by period. Bars show observed proportions; error bars show exact 95% binomial confidence intervals.

Antibiotic exposure during the index gastroenteritis episode was documented in 17/30 (56.7%) pre-pandemic cases, 5/39 (12.8%) pandemic cases, and 23/69 (33.3%) post-emergency cases (χ^2^ = 14.87, df = 2, *p* = 0.000591; false-discovery-rate-adjusted *p* = 0.00236; Cramér V = 0.33; Table 3 and Figure 3). Compared with the pandemic period, exposure was 43.8 percentage points higher pre-pandemic (95% CI 21.5–61.4; Holm-adjusted *p* = 0.00055) and 20.5 points higher post-emergency (95% CI 3.4–34.3; Holm-adjusted *p* = 0.045). Pre-pandemic exposure exceeded post-emergency exposure by 23.3 points (95% CI 2.3–42.2; Holm-adjusted *p* = 0.045).

Among 45 antibiotic-exposed patients, 42 (93.3%) received monotherapy and three (6.7%) received combination therapy. Metronidazole was recorded most frequently (*n* = 24), followed by fluoroquinolones (*n* = 9), macrolides (*n* = 8), and beta-lactams (*n* = 4). The median duration was 7 days (IQR 4–8), and 19 exposures preceded the microbiology result. Antibiotic class and duration by period are summarized in Supplementary Table S8. In the exploratory adjusted model, the pandemic (adjusted OR 0.31, 95% CI 0.08–1.25; *p* = 0.100) and post-emergency (adjusted OR 0.63, 95% CI 0.22–1.81; *p* = 0.395) coefficients were not statistically significant relative to the pre-pandemic period. Confirmed enteric pathogens were strongly associated with antibiotic exposure (adjusted OR 16.28, 95% CI 3.03–87.35; *p* = 0.001), while fever, age, and sex were not (Supplementary Table S6).

### 3.4. IBS Subtype and Psychiatric Diagnoses

IBS-M was the most frequent subtype (59/138, 42.8%), followed by IBS-D (52/138, 37.7%) and IBS-C (27/138, 19.6%). Subtype distribution did not differ across periods (χ^2^ = 2.82, df = 4, *p* = 0.588; Cramér V = 0.10; adjusted *p* = 0.876). Any documented psychiatric diagnosis was present in 46/138 (33.3%) of cases and a newly documented post-gastroenteritis diagnosis was present in 24/138 (17.4%); neither differed across periods (raw *p* = 0.876 and *p* = 0.667, respectively; adjusted *p* = 0.876 for both; Table 4). These non-significant comparisons should not be interpreted as evidence of equivalence because period-specific samples were modest.

### 3.5. Sensitivity Analyses

The period association remained after excluding all 23 SARS-CoV-2-associated cases (*n* = 115; χ^2^ = 19.34, df = 6, *p* = 0.0036; V = 0.29; Supplementary Table S1), demonstrating that the primary result was not solely attributable to the structural absence of SARS-CoV-2 before the pandemic. Among the 111 cases whose index gastroenteritis and IBS diagnosis occurred within the same period, the five-category association also persisted (χ^2^ = 22.16, df = 8, *p* = 0.0046; V = 0.32; Supplementary Table S2). Results were directionally concordant when all 163 detailed-review records were included (four-category profile: χ^2^ = 32.00, df = 6, *p* < 0.001; V = 0.31) and in the 68 microbiologically confirmed cases (χ^2^ = 28.63, df = 4, *p* < 0.001; V = 0.46; Supplementary Tables S4 and S5).

## 4. Discussion

### 4.1. Principal Findings

In this single-centre case series, the composition of pathogen/testing profiles among patients ultimately classified as having PI-IBS differed substantially across the pre-pandemic, pandemic, and post-emergency periods. Classical bacterial and protozoal exposures represented a much smaller proportion of diagnosed cases during the pandemic, SARS-CoV-2-associated cases emerged, and the proportion of untested cases increased. After the emergency period, bacterial and protozoal exposures partially reappeared while SARS-CoV-2-associated cases persisted. The association remained when SARS-CoV-2 cases were removed and when analyses were restricted to patients whose gastroenteritis and IBS diagnosis occurred within the same period.

These patterns are compatible with changes reported by enteric-infection surveillance systems. FoodNet documented substantial pandemic-era reductions in reported bacterial enteric infections [5], while international giardiasis notifications fell by more than one third [6]. The partial post-emergency representation of bacterial and protozoal cases is also compatible with broader changes in exposure and susceptibility after relaxation of public health measures [7]. The increased proportion of untested cases during the pandemic indicates that the observed profile cannot be interpreted solely as a change in pathogen circulation. Reduced healthcare attendance, altered clinical thresholds for stool testing, laboratory workload displacement toward SARS-CoV-2 diagnostics, and changes in documentation practices operated concurrently, and none of these can be separated from true changes in pathogen ecology in this design. The pathogen/testing profile should therefore be read as a joint description of what patients were exposed to and how they were investigated, not as a surveillance estimate of pathogen circulation.

### 4.2. SARS-CoV-2-Associated PI-IBS

The appearance of SARS-CoV-2-associated PI-IBS is biologically and clinically plausible. Prospective studies have documented new-onset IBS after COVID-19 [9,10], and meta-analysis has identified a substantial association between SARS-CoV-2 infection and PI-IBS [2]. Prolonged gastrointestinal shedding, viral antigen persistence, and tissue-based immune activation provide potential mechanistic pathways [14,15,16]. Nevertheless, our case series cannot estimate the absolute or relative risk of PI-IBS after SARS-CoV-2 infection because it lacks a denominator of all infected patients and a non-PI-IBS comparison group.

### 4.3. Antibiotic Exposure

Antibiotic exposure also differed markedly in the unadjusted period comparison, paralleling broad reductions in outpatient antibiotic prescribing during the pandemic [8]. However, the adjusted analysis materially qualified this finding: after confirmed enteric pathogens and other covariates were included, period coefficients were no longer statistically significant, whereas confirmed bacterial or protozoal infection was strongly associated with antibiotic exposure. The wide confidence interval reflects the small number of exposures within some strata. Accordingly, our results support a period-associated change in the observed antibiotic-exposure profile among cases, but they do not establish an independent pandemic-related change in prescribing practice. This distinction is important because antibiotics are themselves associated with subsequent PI-IBS in prior literature [3,4].

These adjusted estimates must be interpreted cautiously. With 45 exposed cases and six slope parameters, the model is at the limit of conventional events-per-variable guidance, and the very wide interval around the confirmed-enteric-pathogen estimate (95% CI 3.03–87.35) reflects sparse strata rather than precise effect estimation. Residual confounding is likely: acute-illness severity, treatment indication, care setting, and prescriber-level factors were not retrievable and could not be modelled. Prescribing indication was not recorded in a retrievable form, and fever was the only acute-severity marker available for modelling. Because 19 of 45 exposures preceded the microbiology result, part of the association between confirmed enteric pathogens and antibiotic exposure may reflect clinical severity or presentation patterns that prompted both testing and empirical treatment, rather than result-guided prescribing; the direction of this bias cannot be determined retrospectively. The model therefore serves only to show that the unadjusted period differences in antibiotic exposure are not independent of case mix; it does not identify determinants of prescribing.

### 4.4. IBS Phenotype and Psychiatric Diagnoses

IBS-M and IBS-D predominated, consistent with the usual PI-IBS phenotype [3,17,18], although IBS-C was approximately twice as frequent as the pooled estimate reported in the most recent meta-analysis (19.6% vs. 10.0%) [2]. We did not demonstrate period differences in subtype or documented psychiatric diagnoses. These results differ in scope from population surveys that estimate prevalence changes in the general population [11,12]. A case series assesses the composition of diagnosed cases, not population prevalence. In addition, subtype at diagnosis does not capture within-patient evolution; long-term follow-up after infectious outbreaks has shown substantial phenotype change over time [19]. Psychiatric findings were also limited to diagnoses documented in routine records rather than standardized measures, and both post-infectious states and COVID-19 have been associated with psychiatric morbidity [20].

### 4.5. Clinical Implications and Hypothesis Generation

The practical value of these findings lies in the interpretation of clinical histories and routine records rather than in establishing a change in treatment. First, one third of pandemic-period cases had no recorded stool microbiology. In such records, pathogen status should be considered unknown rather than microbiologically negative; absence of a recorded test does not establish whether testing was not performed or whether its documentation was unavailable. Second, SARS-CoV-2-associated index illnesses remained represented after the public health emergency (13.0% of post-emergency cases). Together with prior prospective evidence [9,10], this supports including previous COVID-19 accompanied by gastrointestinal symptoms in the infectious history of patients evaluated for possible PI-IBS, without treating temporal association as proof of causation.

Third, confirmed bacterial or protozoal pathogen status was associated with antibiotic exposure in the exploratory adjusted model, whereas calendar-period coefficients were not statistically significant. However, 19 antibiotic exposures preceded microbiology results; this association therefore does not demonstrate that prescribing was guided by those results or establish treatment appropriateness. These observations generate the testable hypothesis that temporal differences in reported PI-IBS pathogen profiles may be partly shaped by changes in microbiological ascertainment and case mix. Future prospective studies should record test ordering, test results, and treatment timing separately and evaluate pathogen-specific outcomes while accounting for testing patterns.

All three implications concern the interpretation of individual clinical histories and routine records. None of them implies that PI-IBS became more or less frequent in any period: this series measures the composition of diagnosed cases, and only denominator-based prospective designs can measure incidence. Where our composition findings and population-survey prevalence findings appear to diverge, the two designs are measuring different quantities rather than contradicting each other.

### 4.6. Strengths and Limitations

Strengths include a clearly defined period-based question, separation of tested-negative from untested records, effect-size reporting, multiplicity control, a fixed-margin Monte Carlo assessment for the primary table, and several sensitivity analyses addressing the structural SARS-CoV-2 zero and the lag between gastroenteritis and IBS diagnosis. The use of the same institutional records across periods reduced between-centre heterogeneity.

The limitations are substantial. First, this is a case series without a denominator of all acute gastroenteritis episodes or a non-IBS control group. It therefore cannot estimate PI-IBS incidence, relative risk, or causal effects, and period-specific case counts must not be read as incidence trends. Second, calendar period is a composite exposure that bundles pathogen circulation, public health measures, healthcare access, testing, and prescribing. Third, formal Rome IV components were not prospectively measured. Aggregate reasons for the 25 exclusions after detailed re-adjudication were available (recurrent abdominal pain criterion not met, *n* = 7; insufficient associated bowel-habit criteria, *n* = 5; chronicity criterion not met, *n* = 6; insufficient documentation, *n* = 7), but exact symptom-onset dates, item-level pain-frequency data, and Bristol Stool Form Scale percentages were unavailable. Fourth, diarrhea and vomiting were documented in every detailed-review record, which may reflect standardized recording conventions and could overstate the completeness of acute-feature ascertainment. Fifth, stool-test ordering variables contributed directly to the operational tested-negative/not-tested categories and could not be interpreted as independent outcomes. Moreover, no SARS-CoV-2-associated case had stool microbiology recorded during the index illness, so concurrent enteric infection could not be microbiologically excluded in these 23 cases. Sixth, psychiatric diagnoses came from routine clinical documentation rather than standardized instruments, and granular acute-illness severity and proton-pump inhibitor exposure were not consistently retrievable. Prescribing indication was likewise not retrievable, and undocumented pre-existing bowel symptoms before the index episode cannot be fully excluded. Seventh, the delay between index gastroenteritis and IBS diagnosis created period crossover in 27 cases, although a same-period sensitivity analysis yielded concordant findings. Eighth, the three periods differed in calendar length (14, 39, and 19 months) and in post-episode ascertainment time: unequal length affects the absolute number of cases accrued per period—which we do not interpret—and can also influence case mix if pathogen circulation or care practices varied within a period; late-2024 gastroenteritis episodes additionally had less opportunity to generate a diagnosis before the record review was completed, potentially under-representing the latest post-emergency months. Finally, small cells and the single-centre retrospective design limit precision and generalizability.

## 5. Conclusions

Among chart-adjudicated PI-IBS cases at one tertiary centre, pathogen/testing composition and the observed antibiotic-exposure profile differed across the pre-pandemic, pandemic, and post-emergency periods. The primary association persisted after excluding SARS-CoV-2 cases and after restricting analysis to patients whose gastroenteritis and IBS diagnosis occurred within the same period. These findings describe how diagnosed-case composition changed; they do not establish changes in PI-IBS incidence or risk and cannot separate infection ecology from diagnostic behaviour. For clinicians and researchers, an index gastroenteritis episode with no recorded stool microbiology should be regarded as having unknown pathogen status, not as a microbiologically negative episode. Denominator-based prospective cohorts with standardized Rome IV symptom assessment, acute-illness severity measures, microbiological testing, and psychological instruments are required.

## Figures and Tables

**Table 1:** Baseline characteristics of the primary chart-adjudicated PI-IBS cohort by index gastroenteritis period.

Characteristic	Pre-Pandemic (*n* = 30)	Pandemic (*n* = 39)	Post-Emergency (*n* = 69)	*p* Value
Female sex, *n* (%)	20 (66.7)	28 (71.8)	45 (65.2)	0.779
Age, years, mean ± SD	41.2 ± 13.7	40.3 ± 17.5	40.8 ± 12.5	0.802
Gastroenteritis-to-diagnosis interval, days, median (IQR)	280 (259–293)	276 (254–304)	276 (260–299)	0.892
Fever documented, *n* (%)	20 (66.7)	9 (23.1)	34 (49.3)	0.001
Diabetes mellitus, *n* (%)	2 (6.7)	4 (10.3)	9 (13.0)	0.638
Hypertension, *n* (%)	5 (16.7)	5 (12.8)	16 (23.2)	0.393
Antidepressant use, *n* (%)	7 (23.3)	8 (20.5)	15 (21.7)	0.961
NSAID use, *n* (%)	10 (33.3)	13 (33.3)	29 (42.0)	0.574

Values are based on the date of the index gastroenteritis episode. *p* values are descriptive and unadjusted; continuous variables were compared with Kruskal–Wallis tests and categorical variables with Pearson chi-squared or Fisher exact tests. IQR, interquartile range; NSAID, non-steroidal anti-inflammatory drug; PI-IBS, post-infectious irritable bowel syndrome; SD, standard deviation.

**Table 2:** Five-category pathogen/testing profile by index gastroenteritis period (primary comparison).

Category	Pre-Pandemic (*n* = 30)	Pandemic (*n* = 39)	Post-Emergency (*n* = 69)	Total
Bacterial, *n* (%)	10 (33.3)	1 (2.6)	10 (14.5)	21
Protozoal, *n* (%)	9 (30.0)	2 (5.1)	13 (18.8)	24
SARS-CoV-2, *n* (%)	0 (0.0)	14 (35.9)	9 (13.0)	23
Tested negative, *n* (%)	7 (23.3)	9 (23.1)	23 (33.3)	39
Not tested, *n* (%)	4 (13.3)	13 (33.3)	14 (20.3)	31

Pearson χ^2^ = 35.56, df = 8, asymptotic *p* = 0.000021; fixed-margin Monte Carlo *p* = 0.000030 (200,000 random tables); Cramér V = 0.36, stratified-bootstrap 95% CI 0.30–0.48. “Tested negative” denotes recorded stool microbiology with no identified pathogen; “not tested” denotes no recorded stool microbiology.

**Table 3:** Antibiotic exposure during index gastroenteritis by period and pairwise contrasts.

Comparison	Exposed/Total (%)	Risk Difference, Percentage Points	95% CI	Holm-Adjusted *p*
Pre-pandemic	17/30 (56.7)	—	—	—
Pandemic	5/39 (12.8)	—	—	—
Post-emergency	23/69 (33.3)	—	—	—
Pre-pandemic vs. pandemic	—	43.8	21.5 to 61.4	0.00055
Post-emergency vs. pandemic	—	20.5	3.4 to 34.3	0.045
Pre-pandemic vs. post-emergency	—	23.3	2.3 to 42.2	0.045

Overall Pearson χ^2^ = 14.87, df = 2, *p* = 0.000591; Benjamini–Hochberg-adjusted *p* = 0.00236; Cramér V = 0.33. Pairwise *p* values are from Fisher exact tests with Holm correction. Risk-difference confidence intervals use the Newcombe–Wilson method. CI, confidence interval.

**Table 4:** IBS subtype and documented psychiatric diagnoses by index gastroenteritis period (exploratory comparisons).

Outcome	Pre-Pandemic (*n* = 30)	Pandemic (*n* = 39)	Post-Emergency (*n* = 69)	Raw *p*	BH-Adjusted *p*
IBS-C, *n* (%)	5 (16.7)	7 (17.9)	15 (21.7)	0.588 *	0.876
IBS-D, *n* (%)	9 (30.0)	18 (46.2)	25 (36.2)		
IBS-M, *n* (%)	16 (53.3)	14 (35.9)	29 (42.0)		
Any documented psychiatric diagnosis, *n* (%)	9 (30.0)	14 (35.9)	23 (33.3)	0.876	0.876
New post-gastroenteritis psychiatric diagnosis, *n* (%)	6 (20.0)	5 (12.8)	13 (18.8)	0.667	0.876

* Single overall subtype comparison across the three subtype rows: χ^2^ = 2.82, df = 4, *p* = 0.588; Cramér V = 0.10. The Benjamini–Hochberg family comprised antibiotic exposure, subtype, any psychiatric diagnosis, and new post-gastroenteritis psychiatric diagnosis. BH, Benjamini–Hochberg; IBS-C, IBS with constipation; IBS-D, IBS with diarrhea; IBS-M, mixed IBS.

## Data Availability

The de-identified analytic dataset supporting the findings is available from the corresponding author upon reasonable request, subject to institutional policy and ethical data-protection requirements.

## Supplementary Materials

The following supporting information can be downloaded at: https://www.mdpi.com/article/10.3390/jcm15197729/s1, Table S1: pathogen/testing profile after exclusion of SARS-CoV-2-associated cases; Table S2: analysis restricted to cases whose index gastroenteritis and IBS diagnosis occurred within the same period; Table S3: four-category confirmation analysis in the primary cohort; Table S4: four-category data-integrity sensitivity analysis including all 163 detailed-review records; Table S5: pathogen distribution among microbiologically confirmed primary-cohort cases; Table S6: exploratory multivariable logistic regression for antibiotic exposure; Table S7: distribution of individual organisms by period; Table S8: antibiotic class and duration by period.
