# Supplementary material for: Pathogen and Testing Profiles in Post-Infectious Irritable Bowel Syndrome Across the COVID-19 Public Health Emergency: A Retrospective Case Series
Source: J Clin Med. 2026 Oct 8;15(19):7729. doi: 10.3390/jcm15197729 (PMC13666457; doi:10.3390/jcm15197729)
Supplement: Supplementary file 1 [file jcm-15-07729-s001.zip › jcm-4578200-supplementary.pdf]

Supplementary Materials

Pathogen and Testing Profiles Among Post-Infectious Irritable Bowel Syndrome Cases Before, During, and After the COVID-19 Public Health Emergency: A Retrospective Case Series

Journal of Clinical Medicine submission

Supplementary Statistical Methods

The primary Pearson chi-squared result was supplemented by a fixed-margin Monte Carlo test based on 200,000 random contingency tables. The Cramér V confidence interval was obtained by stratified resampling within the three index-gastroenteritis periods (10,000 bootstrap samples). Pairwise antibiotic-exposure contrasts used Fisher exact tests with Holm correction and Newcombe-Wilson confidence intervals for absolute risk differences. All analyses were complete-case analyses of the variables displayed.

Supplementary Table S1. Pathogen/testing profile after exclusion of SARS-CoV-2-associated cases (*n* = 115).

Category	Pre-Pandemic (*n* = 30)	Pandemic (*n* = 25)	Post-Emergency (*n* = 60)	Total
Bacterial	10 (33.3)	1 (4.0)	10 (16.7)	21
Protozoal	9 (30.0)	2 (8.0)	13 (21.7)	24
Tested negative	7 (23.3)	9 (36.0)	23 (38.3)	39
Not tested	4 (13.3)	13 (52.0)	14 (23.3)	31

Pearson $\chi^2 = 19.34$, $df = 6$, $p = 0.0036$; *Cramér* $V = 0.29$.

Supplementary Table S2. Five-category profile restricted to cases whose index gastroenteritis and IBS diagnosis occurred within the same period (*n* = 111).

Category	Pre-Pandemic (*n* = 14)	Pandemic (*n* = 28)	Post-Emergency (*n* = 69)	Total
Bacterial	5 (35.7)	1 (3.6)	10 (14.5)	16
Protozoal	4 (28.6)	2 (7.1)	13 (18.8)	19
SARS-CoV-2	0 (0.0)	9 (32.1)	9 (13.0)	18
Tested negative	3 (21.4)	5 (17.9)	23 (33.3)	31
Not tested	2 (14.3)	11 (39.3)	14 (20.3)	27

Pearson $\chi^2 = 22.16$, $df = 8$, $p = 0.0046$; *Cramér* $V = 0.32$.

Supplementary Table S3. Four-category confirmation analysis in the primary cohort, combining tested-negative and untested records (*n* = 138).

Category	Pre-Pandemic (*n* = 30)	Pandemic (*n* = 39)	Post-Emergency (*n* = 69)	Total
Bacterial	10 (33.3)	1 (2.6)	10 (14.5)	21
Protozoal	9 (30.0)	2 (5.1)	13 (18.8)	24
SARS-CoV-2	0 (0.0)	14 (35.9)	9 (13.0)	23
Negative/untested	11 (36.7)	22 (56.4)	37 (53.6)	70

Pearson $\chi^2 = 32.52$, $df = 6$, $p < 0.001$; *Cramér* $V = 0.34$.

Supplementary Table S4. Four-category data-integrity sensitivity analysis including all 163 detailed-review records irrespective of the final Rome IV-compatible flag.

Category	Pre-Pandemic (*n* = 35)	Pandemic (*n* = 50)	Post-Emergency (*n* = 78)	Total
Bacterial	12 (34.3)	3 (6.0)	11 (14.1)	26
Protozoal	9 (25.7)	5 (10.0)	14 (17.9)	28

Category	Pre-Pandemic (n = 35)	Pandemic (n = 50)	Post-Emergency (n = 78)	Total
SARS-CoV-2	0 (0.0)	18 (36.0)	10 (12.8)	28
Negative/untested	14 (40.0)	24 (48.0)	43 (55.1)	81

Pearson $\chi^2 = 32.00$, $df = 6$, $p < 0.001$; Cramér V = 0.31. Records classified as not Rome IV-compatible are not PI-IBS cases; this analysis evaluates robustness of the period-profile signal only.

Supplementary Table S5. Pathogen distribution among microbiologically confirmed primary-cohort cases (n = 68).

Category	Pre-Pandemic (n = 19)	Pandemic (n = 17)	Post-Emergency (n = 32)	Total
Bacterial	10 (52.6)	1 (5.9)	10 (31.2)	21
Protozoal	9 (47.4)	2 (11.8)	13 (40.6)	24
SARS-CoV-2	0 (0.0)	14 (82.4)	9 (28.1)	23

Pearson $\chi^2 = 28.63$, $df = 4$, $p < 0.001$; Cramér V = 0.46.

Supplementary Table S6. Exploratory multivariable logistic regression for antibiotic exposure during index gastroenteritis (n = 138; 45 exposed cases).

Variable	Adjusted OR	95% CI	p Value
Pandemic vs pre	0.31	0.08-1.25	0.100
Post-emergency vs pre	0.63	0.22-1.81	0.395
Confirmed enteric pathogen	16.28	3.03-87.35	0.001
Fever	0.53	0.10-2.75	0.454
Age, per year	0.97	0.94-1.01	0.108
Male sex	1.57	0.60-4.11	0.360

Reference period: pre-pandemic. Confirmed enteric pathogen denotes a bacterial or protozoal pathogen. The model contained six slope parameters and is hypothesis-generating; confidence intervals are wide because of sparse exposure in some strata. CI, confidence interval; OR, odds ratio.

Supplementary Table S7. Distribution of individual organisms among primary-cohort cases by index-gastroenteritis period.

Organism	Pre-Pandemic (n = 30)	Pandemic (n = 39)	Post-Emergency (n = 69)	Total
Salmonella spp., n	5	0	4	9
Campylobacter spp., n	2	1	4	7
Shigella spp., n	3	0	2	5
Giardia lamblia, n	8	1	10	19
Entamoeba histolytica, n	1	1	3	5
SARS-CoV-2, n	0	14	9	23

Counts are descriptive. Organism-level cells are too small for inferential comparison; no Salmonella or Shigella infection was documented during the pandemic period.

Supplementary Table S8. Antibiotic class and duration by period among the 45 antibiotic-exposed patients.

Variable	Pre-Pandemic (n = 17)	Pandemic (n = 5)	Post-Emergency (n = 23)
Primary agent: metronidazole, n	8	4	12
Primary agent: fluoroquinolone, n	4	0	5

Primary agent: macrolide, n	4	0	4
Primary agent: beta-lactam, n	1	1	2
Combination therapy, n	1	1	1
Duration, days, median (IQR)	7 (4–7)	6 (6–7)	7 (4.5–9)

Descriptive only; cells are small, particularly in the pandemic period. Class counts refer to the primary recorded agent, with one class per patient, so the class rows sum to the number of exposed patients in each period. Three patients (one per period) received combination therapy; all three combination regimens were metronidazole-based, and the class of the companion agent was not separately recorded in the dataset. Nineteen of 45 exposures preceded the microbiology result.
